# Efficacy of Functional Re-Education as a Treatment for Infantile Flexible Flatfoot: Systematic Review

**DOI:** 10.3390/children12010008

**Published:** 2024-12-24

**Authors:** Cristina Molina-García, George Banwell, Francisco Álvarez-Salvago, Andrés Reinoso-Cobo, Clara Pujol-Fuentes, Jose Medina-Luque, Laura Ramos-Petersen

**Affiliations:** 1 Faculty of Physiotherapy, Podiatry and Occupational Therapy, Catholic University San Antonio-UCAM, 30107 Murcia, Spain; cmolina799@ucam.edu; 2 Nursing and Podiatry, Facultad de Ciencias de la Salud, University of Málaga, 29071 Málaga, Spain; gbanwell@uma.es (G.B.); andreicob@uma.es (A.R.-C.); lauraramos.94@uma.es (L.R.-P.); 3 Instituto de Investigación Biomédica de Málaga (IBIMA), University of Málaga, 29071 Málaga, Spain; 4 Department of Physiotherapy, Faculty of Health Sciences, European University of Valencia, 46010 Valencia, Spain; clara.pujol@universidadeuropea.es; 5 Translational Brain Research, German Centre for Neurodegenerative Diseases (DZNE), 81377 Munich, Germany; jose.med.luque@gmail.com

**Keywords:** flexible flatfoot, pediatrics, children, exercises, functional re-education, stretching, strengthening, corrective exercises

## Abstract

Background: Flexible pediatric flatfoot is an anatomical presentation of the foot that is common in children, and its functional impact raises long-term uncertainty. Functional re-education includes strengthening and stretching exercises for the intrinsic and extrinsic musculature of the foot, proposed as an effective conservative treatment. However, to date, there is no systematic review examining its effectiveness in the pediatric population. This systematic review aims to evaluate the effectiveness of functional re-education as a conservative treatment for flexible pediatric flatfoot, determining which exercises are most effective. Methods: A search (PROSPERO: CRD42023391030) was conducted across six databases, resulting in an initial total of 327 studies. Of these, 11 randomized controlled trials (RCTs) met the inclusion criteria, resulting in a sample of 419 children aged 6 to 14 years with a diagnosis of flexible flatfoot. The evaluated studies present variations in diagnostic criteria, types of exercises, and treatment duration. Results: The results indicate that functional re-education is effective in improving the symptomatology and functionality of the foot in children. In particular, exercises targeting the intrinsic musculature proved to be among the most effective treatments, improving the structural development of the medial longitudinal arch. The reviewed literature recommends a minimum treatment duration of eight weeks. Conclusions: Functional re-education represents an effective conservative treatment option for flexible flatfoot in children, positioning it as the treatment of choice for this condition.

## 1. Introduction

Ninety percent of pediatric podiatric clinic visits are related to flat feet (FF) [1]. In the adult population, the prevalence of FF ranges from 2% to 23% [2]. Among adults with FF, 77% report experiencing back or lower limb pain [3]. For this reason, pediatric flatfoot (PF) has significant clinical implications, and early intervention can minimize its long-term impact. Additionally, for patients with symptomatic PF, the literature highlights a decrease in quality of life, painful symptoms, increased fatigue during physical activity, and reduced motor agility [4]. Various studies supports that FF and misalignment can eventually lead to other pathologies in the foot, ankle, and adjacent structures, either in the near or distant future. They have also revealed that adolescents and adults with FF are twice as likely to experience knee and back pain compared to those without this condition. In addition, an association has been observed between FF and conditions such as plantar fasciopathy, Achilles and posterior tibial tendinopathy, hallux limitus and rigidus, chondromalacia patellae, and patellofemoral pain syndrome. They have also linked FF to hip problems, an increased risk of ankle sprains, and even metatarsal stress fractures [1,2,3,4]. Therefore, if the FF is not treated or corrected, it could cause many problems in the long run.

There is no universally agreed-upon definition of PF, although it is typically identified when there are more than two signs, symptoms, or positive test results present [5]. Clinically, PF is characterized by a reduction in the medial longitudinal arch (MLA) of the foot, a condition that may be corrected when the child is non-weight-bearing or through specific maneuvers if the PF is flexible [6]. Since it is a triplanar deformity, FF may also involve forefoot abduction relative to the hindfoot, a valgus position of the calcaneus, a more prominent medial talar head, and internal rotation of the tibia [7,8]. Although there are various diagnostic tests available, the most commonly used for diagnosing FF are the Heel Rise Test, Jack’s Test, Navicular Drop Test (NDT), Navicular Height, Resting Calcaneal Stance Position (RCPS), Foot Posture Index (FPI-6), and Arch Index [5,7,8,9]. In summary, we find that, from a clinical perspective, PF is characterized by a reduction in the ALI when the individual is in a standing or carrying position. This general feature is associated with a valgus position of the calcaneus or hindfoot exceeding 6 degrees (equinus and valgus deviation of the calcaneus). Medial prominence of the talus is noted, the footprint shows a flat appearance, and the ASA tends to be medialized. In addition, there is an ABD of the forefoot in relation to the rearfoot, and internal rotation of the tibia can be identified. All of these features contribute to giving the foot a flattened appearance [5,6,7,8,9,10].

The foot’s active support system includes both the extrinsic and intrinsic muscles. The extrinsic muscles are the primary movers of the foot, while the intrinsic muscles play a key role in stabilizing the MLA. The extrinsic muscles provide dynamic support to the arch during gait, while the intrinsic muscles regulate the stiffness of the MLA in both static and dynamic contexts [11,12]. The posterior tibialis (TP), a key extrinsic muscle, supports the MLA by contracting eccentrically during the stance phase of gait to control arch flattening. It also assists in foot adduction, supination, and flexion [13].

Research indicates that the MLA is supported by a complex structure of ligaments, tendons, and joint capsules that together contribute to the foot’s stability and functionality [14]. When the MLA is reduced or absent, the foot loses its optimal shock-absorbing capacity, potentially leading to various pathologies. This loss of cushioning can increase the risk of deformities such as hallux abductor valgus [15], as well as patellofemoral pain, medial tibial stress syndrome, and lower back pain [16]. Moreover, FF is associated with conditions like plantar fasciitis, Achilles tendinopathy, and pain during weight-bearing [17,18,19]. Therefore, FF not only affects the foot’s structural integrity but also predisposes the joints and muscles of the lower limbs to injury, negatively impacting the quality of life of affected individuals [19,20]. In severe cases, it can compromise the ability to walk and perform daily activities [21]. These factors underscore the importance of early assessment and management of FF to prevent long-term complications and improve patients’ quality of life.

The neural subsystem of the ankle–foot complex includes sensory receptors within the fascia, capsules, and ligaments, which provide the brain with information on the position and movement of the foot. This feedback helps coordinate muscle activity to maintain the MLA [10,22]. Since the muscles are responsible for supporting the MLA, we propose that functional training (FT) of these muscles is essential for improving the signs and symptoms of pediatric flexible flatfoot (PFF).

One contributing factor to the flattening of the MLA in weight-bearing positions is insufficient strength in the foot muscles [23]. Studies have shown that children who engage in less physical activity are more likely to develop FF [23]. Inadequate physical activity can lead to delayed or uneven muscle strength development, resulting in a weakened MLA. Regular exercise is closely associated with physical development, weight control, and overall health [24].

Conservative treatments for PFF are numerous (none of them present sufficient scientific evidence), with foot orthoses (FO) being among the most commonly used. However, these treatments often lead to increased complaints from children and their parents, such as anxiety, footwear restrictions, social stigma, initial discomfort, and the cost of the devices [25,26]. Among all available treatments, exercise plays a crucial role, whether through barefoot walking or exercises to strengthen or stretch muscles that affect the FF deformity [25]. Exercise therapy improves foot muscle strength, providing dynamic support and aiding in the stabilization and maintenance of the MLA [27]. The literature widely supports the effectiveness of strengthening foot function through therapeutic exercise programs for individuals with FF.

Exercise programs generally focus on lengthening tight structures, strengthening weaker areas, and improving proprioception and postural balance [9]. Muscle exercise and strengthening are considered potential first-choice treatments for normal foot development, though the evidence for this remains insufficient, particularly in pediatric populations [28].

Conservative treatments, such as exercise, are increasingly preferred due to their effectiveness in treating various conditions like plantar fasciopathy, TP dysfunction, and Achilles tendinopathy [29,30], as well as systematic reviews on FF treatment [31,32,33,34], which show no adverse effects, greater accessibility, cost-effectiveness, and significant benefits. However, none of these reviews included pediatric populations. Therefore, if early treatment for PFF is not initiated, the likelihood of clinical complications increases over time. This highlights the need for an active, non-invasive, and affordable treatment approach [35]. Consequently, this review aims to assess the effectiveness of functional rehabilitation (FR) in pediatric flexible flatfoot (PFF), specifically evaluating strengthening exercises, stretching, and lower limb exercises as part of treatment, while identifying the most effective exercises and the minimum duration required for improvement.

It is hypothesized that “FR, which includes strengthening, stretching, and lower extremity exercises, effectively improves the signs and symptoms of PFF, with the minimum duration of treatment and type of RF employed being key factors in achieving significant clinical improvement in children with PFF”.

## 2. Materials and Methods

This systematic review was registered with the International Prospective Register of Systematic Reviews PROSPERO under the number CRD42023391030. To address the objectives outlined in this study, a systematic review was conducted following the “Preferred reported items of systematic reviews and meta-analysis” regulations (PRISMA), as well as the recommendations of the Cochrane Collaboration [36]. For detailed information regarding the PRISMA checklist, refer to Supplementary Table S1 (PRISMA checklist).

### 2.1. Selection Criteria

*Types of studies*

Published randomized controlled clinical trials (RCCTs) were included. Other types of studies, including systematic reviews, were not considered.

No filters for publication, such as year or language, were applied in order to avoid restricting the search.

In summary, the systematic review focused on including only RCCTs, with no restrictions on language or year of publication, in order to obtain a broad and complete view of the available evidence.

*Participants*

The studies reviewed specifically focused on children who were diagnosed with PFF. The age of the participants had to be 12 years or younger (so that the foot was not yet fully formed). Patients who had undergone lower limb surgery or had a systemic or infectious neurological condition were excluded.

*Type of intervention*

Studies that considered functional rehabilitation (strengthening, stretching, foot, or lower limb exercises) as primary treatments were included. Additionally, each study had to include at least two groups to enable comparison of the exercise effects.

*Comparison*

Studies that include another type of conservative treatment, such as other types of functional rehabilitation or placebo.

*Outcome measure*

The outcomes included were those that evaluated changes in the signs and symptoms of PFF, such as MLA formation, reduction in painful symptomatology, improvements in test scores, among others.

### 2.2. Search Strategy

Two researchers (C.M.-G and L.R.-P) carried out the search independently in the following databases: PubMed, EBSCO, Web of Science, Cochrane, SCOPUS, and PEDro. In addition, the references from the included papers were reviewed. The last search was carried out in September 2024.

The Medical Subject Headings (MeSHs) that were used were as follows: flatfoot, pediatrics, child, exercise, according to the characteristics of each database, accompanied by the Boolean operators “AND” and “OR”.

The following search strategy was used: ((“Flatfoot”[Mesh] AND (“Pediatrics”[Mesh] OR “Child”[Mesh] OR “Child, Preschool”[Mesh] OR “Infant”[Mesh])) AND (“Exercise Therapy”[Mesh] OR “Exercise”[Mesh])).

The following search strategy was also used to include free terms and Thesaurus synonyms: (((“Flatfoot”[Mesh] AND (“Pediatrics”[Mesh] OR “Child”[Mesh] OR “Child, Preschool”[Mesh] OR “Infant”[Mesh])) AND (“Exercise Therapy”[Mesh] OR “Exercise”[Mesh])) OR (((“Flexible Flatf**t”[tw] OR “Flat F**t”[tw] OR “Pes Planus”[tw] OR Flatf**t[tw] OR Splayfoot[tw] OR “F**t, Flat” [tw] OR “Flatf**t, Flexible”[tw]) AND (“Pediatrics”[tw] OR “Child”[tw] OR “Infant”[tw] OR “Preschool Child*”[tw] OR “Child*, Preschool”[tw])) AND (“Exercise Therapy”[tw] OR “Exercise”[tw] OR “muscle strenght”[tw] OR “stretching”[tw]) OR “strengthening”[tw] OR “functional reeducation”[tw] OR “rehabilitation”[tw])).

### 2.3. Study Selection

Two researchers (C.M.-G and L.R.-P) carried out the selection of the studies. After searching in the databases, the duplicates were eliminated. After that, the titles and abstracts were screened. Then, the studies were fully read to be selected. Another reviewer (G.B) was consulted if there were any disagreements.

### 2.4. Data Extraction and Management

To respond to the presented objectives, some data were extracted from the studies: characteristics of the publication (author, year, country, study design), characteristics of the sample (sample size, age, year, gender, weight, height, body mass index, previous treatment, diagnosis, symptoms and setting), characteristics of the intervention (type of exercise, exercise protocol, frequency and duration of the treatment, supervision, and outcome measure to assess the intervention), characteristics of the diagnosis, and the results together with the final conclusions.

### 2.5. Risk of Bias and Quality Assessment

To assess the methodological quality and the risk of bias of the included studies, some scales were used for the study types RCCTs.

The Cochrane Collaboration’s Risk of Bias 2.0 tool was employed to assess the methodological rigor of included RCTs. This involved evaluating bias across five domains: randomization process, deviations from intended interventions, missing outcome data, measurement of outcomes, and selective reporting. Each domain was graded as “low risk”, “some concerns”, or “high risk” [37]. In addition, the Scottish Intercollegiate Guidelines Network (SIGN) scale was used to reflect the level of evidence and degree of recommendation [38].

### 2.6. Data Synthesis

Tables and narrative forms were used to describe the characteristics of the studies. It was impossible to perform a meta-analysis because the studies were not sufficiently homogeneous.

## 3. Results

**Figure 1:**
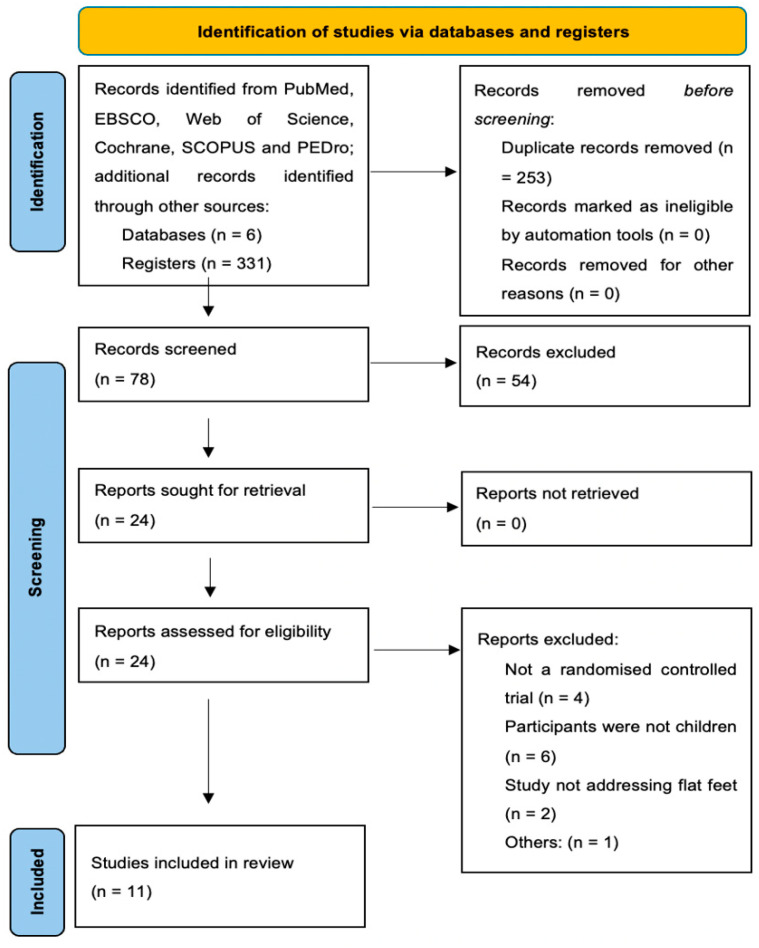
PRISMA flow diagram.

Using the search strategy outlined above, we identified a total of 327 studies in the databases, along with two additional records obtained from other sources, specifically the reference lists of the initial papers. Of these 331 records, 253 were duplicates. The remaining 78 studies were reviewed by title and abstract by two independent reviewers. Of these, 54 were excluded due to differences in inclusion criteria, as they were observational studies, clinical trials involving a single group, or did not involve children as participants. Subsequently, 24 full-text articles were assessed for eligibility, and 13 were excluded for reasons such as participants having previously undergone lower limb surgery or unspecified exercises, among others. Therefore, only 11 RCTs fully met the inclusion criteria. The PRISMA flow diagram for the studies included in this review is shown in Figure 1.

### 3.1. General Characteristics of the Assessed Studies

Studies from multiple countries were included: Iran, Poland, South Korea, India, Romania, Egypt, Saudi Arabia, and Indonesia, published between 2016 and 2024. The oldest study meeting our inclusion criteria was published by Khamooshi et al. [39] in 2016, while the most recent study was by Ketabchi et al. [40] in 2024. Regarding evidence levels assessed, the included studies presented II + A and II + B levels, after using the SIGN grading system, indicating quasi-experimental designs or controlled clinical trials.

Gender distribution varied across studies; some studies included only boys or girls, while others included both. Of the 419 participants included, 162 were girls and 147 boys. Gender information for the remaining 110 subjects was unavailable, as the authors did not provide it. Mean ages also varied, ranging from 6 to 14 years, with an average age of 9–10 years. Priyanka et al. [41] did not provide specific ages for subjects, though they indicated the children were in the 8th and 9th grades, leading us to estimate their ages as 13–14 years old. The group sizes varied across the studies, with sample sizes ranging from 9 to 36 children per group. The study by Abd-Elmonem et al. [42] had the largest sample size with 72 subjects.

In terms of participant characteristics, the average body mass index (BMI) was 22.19. Some studies did not provide BMI data [39,40,41,43,44]. All included participants were required to be diagnosed with flatfoot (FF); however, each author used different terms to describe the diagnosis. For example, Rusu et al. [45] used a more detailed term “bilateral flexible flatfoot level II asymptomatic.” Only four authors [39,40,46,47] reported that the participants in their study had not received prior treatments. Regarding symptomatology, Abd-Elmonem et al. [42], Rusu et al. [45], and Ketabchi et al. [40] indicated that the FF was asymptomatic. The only author reporting symptomatic FF in their participants was Markowicz et al. [47] (Table 1).

The studies primarily included children with FF, and some authors also considered characteristics such as weight [46,48] or symptomatology [40,42,47,48]. Subjects were excluded if they had undergone lower limb surgeries, had neuromuscular, neurological, or hereditary diseases, or had fractures or injuries 3–6 months before the study started. Moreover, some authors specifically excluded subjects who were unwilling to participate in the study or due to the sport they practiced [44,46], or had foot pain or rigid FF [40,48].

The methods for evaluating flexible FF varied considerably among previous studies. Each author used different criteria to diagnose pediatric FF. The navicular drop test (NDT) was the most frequently used test, being part of 5 [39,40,42,46,48] of the 12 included articles. This test measures the vertical displacement of the navicular bone from a seated, non-weight-bearing position to a standing, weight-bearing position. A significant drop indicates potential flatfoot, although each author applied different values to determine a positive or negative result. In contrast, Priyanka et al. [41] used the Navicular Height variant, which measures the height of the navicular bone above the ground while standing, emphasizing structural assessment over dynamic changes. Meanwhile, Khamooshi et al. [39] employed the navicular collapse test, a variation that evaluates the extent to which the navicular bone moves medially or “collapses” during weight-bearing activities. The Arch Height Index was the second most frequently used test [39,43,44,45,49]. This index assesses the ratio of the height of the medial arch (measured at the highest point of the arch) to the length of the foot. None of the authors used radiographic examination for diagnosis, which is typically considered the gold standard for structural foot evaluation in clinical settings. The exclusion of this method reflects the focus on non-invasive and functional assessments in these studies.

Additionally, the following tests were identified in the various included articles:

- The foot posture index (FPI-6) evaluates the overall alignment of the foot using six clinical criteria, such as the position of the talar head, arch curvature, and calcaneal alignment, providing a comprehensive and multi-dimensional assessment of foot posture.
- The Too Many Toes test assesses foot alignment by observing how many toes are visible from a posterior view when the patient stands. A greater number of visible toes indicates pronation and potential flatfoot.
- The Resting Calcaneal Stance Position (RCPS) measures the angle of the calcaneus in a resting standing position to determine whether it is in valgus, which is often associated with flatfoot.
- The Staheli Index uses footprints to calculate the ratio of the narrowest part of the arch to the widest part of the forefoot. A higher ratio suggests a flatter foot.

Only Gheitasi et al. [46] considered foot function and dysfunction in adjacent joints, such as the ankle or knee, highlighting the interconnected nature of lower limb biomechanics. Additionally, only Rusu et al. [45] employed a force platform, which measures the distribution of pressure and force under the foot during standing or walking, providing detailed data on dynamic foot function. Finally, it is notable that more recent articles [44,47] have incorporated systems to evaluate stability or gait, reflecting a growing emphasis on dynamic and functional assessments over purely structural measures. These systems provide insights into the biomechanical behavior of the foot during movement, which is critical for understanding the implications of flatfoot on overall functionality (Table 2).

Risk of Bias Assessment

**Figure 2:**
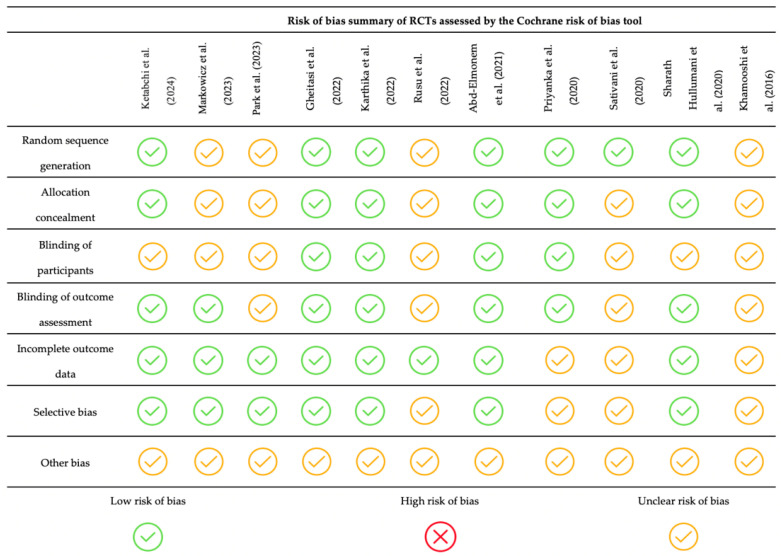
Risk of bias of the included studies. RCT: randomized controlled trial [39,40,41,42,43,44,45,46,47,48,49].

None of the included studies presented a high risk of bias in any domain. The study by Khamooshi et al. [39] showed all items with an unclear risk of bias. The studies by Rusu et al. [45], Sativani et al. [49], and Park et al. [44] had most of their items marked as unclear risk of bias. The remaining included articles generally showed a low risk of bias (Figure 2).

### 3.2. Results by Outcome Measures

As required by our inclusion criteria, there had to be a minimum of two groups to enable comparison of the effect of the exercises. Furthermore, one of the groups had to include functional training (FT) (strengthening, stretching, foot, or lower limb exercises) to more accurately assess the effect of these exercises. Of the 11 included articles, four [39,46,47,49] included a placebo control group, while the remaining studies applied treatments to all groups.

Regarding the type of FT or indicated exercises in each group, a wide variety of exercises was shown. Almost every author implemented different exercises in each group: stretching, intrinsic muscle strengthening, extrinsic muscle strengthening, tibialis posterior strengthening, plyometric exercises, barefoot walking, stretching, exercises combined with neuromuscular electrical stimulation (NMES), plyometric exercises, corrective exercises like toe curls, heel raises, or stability exercises [39,40,41,42,43,44,45,46,47,48,49].

Only Khamooshi et al. [39] included a five-minute warm-up prior to the exercises. Two authors combined exercises with electrotherapy: Abd-Elmonem et al. [42] supplemented one of the exercise groups with 30 min of NMES to enhance intrinsic muscle strengthening, while Priyanka et al. [41] supplemented the exercises with a faradic foot bath administered for 30 min per day. 

All characteristics and descriptions of each exercise performed in the studies are detailed in Table 3.

In terms of exercise duration and frequency, these varied across studies, ranging from 3 days per week in the study by Ketabchi et al. [40] to 5 days per week [43,48], or in the case of Markowicz et al. [47], who included a supervised training session once a week and an unsupervised short foot exercise (SFE) each day for 6 weeks, the average exercise frequency was 3 days per week. There was considerable heterogeneity in frequency due to the variability of exercises, with an average of three sets of 20 repetitions per exercise. Stretching duration ranged from 5 to 15 s per hold. The duration of FT sessions ranged from 30 to 60 min.

Most of the programs were supervised in clinical or rehabilitation settings, with a frequency of 2–3 times per week over periods ranging from 4 to 16 weeks. All exercises were supervised (by physical therapists or researchers), except for the study by Sativani et al. [49], where this information was not provided. The treatment duration ranged from 4 weeks in the studies by Sativani et al. [49] and Priyanka et al. [41] to 16 weeks in the study by Abd-Elmonem et al. [42].

Regarding follow-up of subjects, all the studies conducted two measurements (pre- and post-treatment), except for Priyanka et al. [41], who performed additional measurements (the measurements were taken on the first day before treatment and at the end of the first, second, third, and fourth weeks of treatment), and Ketabchi et al. [40], who conducted mid-treatment measurements (Table 3).

**Table 3:** Intervention characteristics.

Authors	Interventions	Exercise Protocol	Supervision/Treatment Duration (Measurements)
Ketabchi et al. (2024) [40]	CG: Intrinsic foot exercises	The CG focused on intrinsic foot muscle exercises for the first 6 weeks, then switched to extrinsic exercises for the final 6 weeks. Conversely, the IG started with extrinsic exercises and switched to intrinsic exercises for the last 6 weeks. **Intrinsic foot exercises**: The program began with non-weight-bearing exercises (e.g., sitting) and progressed to weight-bearing activities (e.g., standing, single-leg stances) as muscle activation improved. Exercises included short foot exercises, toe spreading, and extensions of the toes. **Extrinsic foot exercise**: The program was emphasized on the posterior tibialis muscle for its role in supporting the MLA, using exercises like foot adduction, heel raises, and foot supination. These were performed initially with minimal resistance, progressing to greater loads with an elastic band and gravity.	Supervised by a professional/12-week corrective exercise program, comprising three 45 min sessions per week. (first, sixth, and twelfth weeks)
	IG: Extrinsic foot exercise		
Markowicz et al. (2023) [47]	CG: None	**Foot intrinsic muscle exercises, without extrinsic muscle acting** (Each exercise consisted of 20 repetitions, which were held for 20 s, with a 20 s rest between repetitions). Each week the exercise changed: - “Foot shortening” and elevating the MLA by drawing the first metatarsal toward the heel, without toe curling. In a seated position, the patient moves the toes while keeping metatarsal heads on the floor and maintaining arch height. “Foot shortening” and MLA elevation, without lifting the toes using feedback stabilization on the knee joints. - “Foot shortening” and MLA elevation, lifting the toes and leaving the metatarsal heads in contact with the floor. Then slowly lowering the toes while maintaining the height of the MLA. - “Foot shortening” and MLA elevation, not lifting the toes off the floor. - “Foot shortening” and MLA elevation, not lifting the toes. The lunge position. Both feet simultaneously. After 20 repetitions, the front limb is changed. - “Foot shortening” and MLA elevation, in the stance phase while walking.	Performed a supervised short foot training session once a week, and an unsupervised SFE each day for 6 weeks; (pre and post treatment)
	IG 1 EBW		
	IG 2 NBW		
Park et al. (2023) [44]	CG: conventional physical therapy	**Conventional physical therapy**: Wearing orthoses, joint mobilization, stretching, and strengthening exercises through weight transfer and weight bearing. **Foot intrinsic muscle strengthening:** Increasing foot awareness using an elastic band.	Supervised in the hospital; 30 min, twice a week for a total of 8 weeks (pre and post treatment)
	IG: foot intrinsic muscle strengthening		
Gheitasi et al. (2022) [46]	CG: None	Intensity/duration: Varies from 10–30 repetitions × 3 **IG 1**: Initial phase (1–4 week): rolling out the feet, toe flexion (curls), big toe extension, lift objects (marble pick-up), short foot (sitting), towel gathering (curls) Improvement phase (5–8 week): toe flexion (curls), big toe extension with resistance, lift smaller objects, standing, towel gathering with weights, toe spread, tennis ball exercise **IG 2:** Initial phase: calf muscle stretch, plantar flexion, hip external rotation, hip abduction, foot adduction, foot supination Improvement phase: calf muscle stretch, plantar flexion with resistance, hip abduction with resistance, hip external rotation, hip extension, foot supination	Supervised in the physical rehabilitation center 3 days per week for 8 weeks (each session: 45–60 min)
	IG1: extrinsic muscles exercises		
	IG 2: intrinsic muscles exercises		
Karthika et al. (2022) [48]	CG: obesity reduction program	**Obesity reduction program**: 30 min moderate intensity aerobic training exercise program 5 days a week for 6 weeks and provided with home-based program sheet/booklet. **Tibialis posterior strengthening exercise program**: 5 days a week for 6 weeks, each session for 30 min. 1- Closed chain resisted foot adduction; 2- Unilateral heel raise (heel raise); 3- Foot supination.	Supervised at the College of Physiotherapy/6 weeks (pre and post treatment)
	IG: obesity reduction program + tibialis posterior strengthening		
Rusu et al. (2022) [45]	CG: Physiotherapand program	**Physiotherapy program** The proposed position was maintained for 10 s followed by 5 s of relaxing; 30 min per session, 3 sessions per week for a total of 12 weeks: Single-leg stance on a fixed surface, forward lean on a fixed surface, standing on one leg on an unstable surface, forward lean on an unstable surface, throwing a ball in different directions on fixed surface, throwing a ball in different directions on an unstable surface, squat on a fixed surface, jump on a fixed surface, squat on an unstable surface and jump on an unstable surface	Supervised in Sports Medicine Department/12 weeks (pre and post treatment)
	IG: Physiotherapand program + foot orthoses		
Abd-Elmonem et al. (2021) [42]	CG: Corrective exercises	- **Corrective exercises** Three times a week, both groups performed 5 strengthening exercises for 60 min with each exercise performed for 30 repetitions holding each repetition for 5 s: Foot intrinsic muscle exercise, exercises with the toes (abduction, extension, and rotation) and selected tibialis posterior muscle strengthening - **Neuromuscular electrical stimulation**: received NMES for 30 min aiming to reinforce the planter intrinsic foot muscles	Supervised in Out-patient Physical Therapy Clinic of Faculty of Physical Therapy/16 weeks (pre and post treatment)
	IG: Corrective exercises + NMES		
Priyanka et al. (2020) [41]	CG: Functional training	**Strengthening Exercises** - Exercises against resistance: Inversion, eversion, and dorsiflexion exercise, with a controlled eccentric return without rotating the leg, gradually increasing the number of sets and repetitions - Double Leg Heel Rises. **Stretching Exercises.**	Physiotherapy/4-week 30 min per day (The measurements were taken very first day prior to treatment and at the end of the first, second, third, and fourth week of the treatment)
	IG: anti-pronation taping + Functional training		
Sativani et al. (2020) [49]	CG: None	Corrective exercises (3 times per week): ◘ Heel lifting (10 repetitions, 3 repetitions) ◘ Toe curls (5 against resistance for 15 s)	N/A/4-week (pre and post treatment)
	IG: corrective exercises		
Sharath Hullumani et al. (2020) [43]	CG: had performed barefoot walking	- Barefoot walking: for 45 min a day barefoot, every day, 5 times a week - Foot-specific exercises, barefoot: 1. Towel gathering exercise for 15 min; 2. Heel cord stretching (holding for 30 s and then relax for 30 s; repeat once); 3. Toe spread (5 s, then 2 s relax); 4. Posterior tibialis exercises (3 sets, 10 repetitions).	Physiotherapist/8 weeks (pre and post treatment)
	IG: had received foot-specific exercises + barefoot walking		
Khamooshi et al. (2016) [39]	CG: None	**Stretching and strengthening exercises**: **The first and second** week of the training were for focusing on the stretching of the Achilles tendon; the long, short, and lateral fibular muscles; the lateral exterior ligaments and the talocalcaneal ligament. **Week 3 and 4** were for strengthening the plantar muscles, tibialis posterior, tibialis anterior, gastrocnemius, soleus, and long flexors of the toes. **Week 5 to 8** of the training program, a combination of the stretching and strengthening movements were carried out. **Exercises related to central stability training,** along with the stretching and strengthening exercises. The central stability-related exercises were carried out in three levels: **Level one** included static contraction exercises, **Level two** incorporated static contraction exercises in an unstable environment, and **Level three** involved dynamic movements in an unstable environment which were carried out by making use of a Swiss ball. At the beginning of every training session, they followed 5 min of warm-up activities, followed by corrective training exercises. The aforesaid exercises took about 25 min during the first sessions, gradually increasing to 45 min during the final sessions. Three times a week, in the form of three sets with 20 repetitions.	Yes/8 weeks (pre and post treatment)
	IG 1: stretching and strength exercises		
	IG 2: stretching and strength exercises + exercises related to central stability training		

Abbreviations: IG: intervention group; CG: control group; MLA: medial longitudinal arch; NBW: normal body weight group; EBW: excessive body weight group; NMES: Neuromuscular electrical stimulation; N/A: Not Available; SFE: short foot exercise.

Different outcome measures were evaluated, although the primary objective in all cases was to assess the effectiveness of the treatment applied for flexible flatfoot (FFF). Some authors focused on morphological changes, such as changes in the FPI-6 [47,48], the NDT [40,42,46], the Foot Print Index [42,47], Staheli Index [39,45], or the Arch Index [43]. In contrast, other authors evaluated foot functionality with tests such as the Star Excursion Balance Test [41], Vertical Jump Height [41], Illinois Agility Test [41], One Leg Stand Test, or the Unterberger Test [49], Postural Stability Test with the Biodex Balance System SD 115VAC [47], and gait parameters assessed with footprints (Gait Velocity (cm/s)); Step Length (cm); Stride Length (cm); Step Angle (°); and Foot Width (cm) [44]. Additional variables included quality of life, pain, or scales such as The Oxford Ankle Foot Questionnaire for Children (OxAFQ-C) [43,49] and the CPA-Questionnaire [48]. Abd-Elmonem et al. [42] also evaluated radiographic changes, including various radiographic indices (anteroposterior and lateral radiographs). Ketabchi et al. [40] was the only author who measured outcomes using ultrasound imaging of foot muscles: abductor hallucis, flexor digitorum brevis and longus, tibialis anterior and posterior (measuring muscle thickness with the probe aligned parallel to muscle fibers).

All authors concluded that FT is effective in improving the signs and symptoms of FFF. Additionally, authors who combined FT with other therapies—such as a weight loss program involving 30 min of aerobic exercise five days a week [48], barefoot walking [43], or NMES [42]—reported greater improvements. Only Priyanka et al. [41] reported similar improvements with FT (strengthening, stretching, and faradic current application) alone as compared to FT combined with anti-pronation taping. Park et al. [44] concluded that strengthening intrinsic foot muscles was more effective than physical therapy combined with orthotic insoles in improving foot morphology and gait ability in children with flatfoot and developmental delays.

Gheitasi et al. [46] concluded that exercise interventions targeting intrinsic and extrinsic foot muscles were feasible, but strengthening intrinsic foot muscles proved more effective than extrinsic exercises for improving flatfoot. These findings align with those of Markowicz et al. [47], who concluded that intrinsic muscle exercises improve foot position and balance. Karthika et al. [48] demonstrated that strengthening the tibialis posterior, alongside weight reduction, not only improves FFF but also enhances the medial longitudinal arch (MLA) and physical activity levels in obese schoolchildren with FFF. Furthermore, Ketabchi et al. [40] concluded that intrinsic muscle exercises are crucial in managing foot flexibility in children, alleviating symptoms and promoting optimized structural and functional foot development.

## 4. Discussion

The aim of this review was to demonstrate the efficacy of FT (strengthening exercises, stretching, or exercises for the foot and lower limbs) as a treatment for pediatric FFF. Additionally, it sought to determine which exercises are most effective for PFF and the minimum duration required to achieve improvements in signs and symptoms.

To address the main objective, studies were analyzed that compared FT exercises with a placebo group or with combinations of FT alongside another therapy or with other types of exercises. This means that, in studies where the comparison group was a placebo [39,46,47,49], the improvements observed in terms of FF signs or symptoms were likely due to the application of FT and not to a natural evolution of the foot. On the other hand, in studies where the comparison group included other therapies or types of exercises [40,41,42,43,44,45,48], improvements can be attributed to the effect of the specific FT exercises implemented. This rejects the hypothesis of many researchers [50,51] who suggest that the improvements found in treatments for pediatric FF are due to the natural evolution of the foot.

This review demonstrates that FT exercises, which may include strengthening exercises, stretching, corrective exercises, plyometric exercises, or barefoot walking, among others, constitute an effective intervention for the treatment of pediatric FFF. These findings are consistent with other recent studies which, although not included in this review due to not meeting the inclusion criteria, also support the effectiveness of RF as the primary treatment in managing pediatric FFF [30,52,53,54,55,56,57,58,59]. It can also be confirmed that this topic has sparked significant scientific interest in recent years. Most of the articles published on this subject are from recent years, with a notable concentration this year [60,61,62,63], focusing on evaluating the effectiveness of RF in treating pediatric FFF. These articles were not included in the review, as they only contained one treatment group; however, their findings are consistent with our results.

Currently, there is no systematic review evaluating the effectiveness of RF or exercises as treatment for FFF in the pediatric population, which is why we believe this is the first review addressing this topic. In contrast, several reviews have been identified that examine this same approach for FFF, but specifically in adults [31,32,33,34,64]. All of them conclude that both RF and exercises are effective in improving the signs and symptoms associated with FFF in the adult population. This year, Cheng et al. [65] published a meta-analysis concluding that exercises targeting the intrinsic muscles of the foot improve both the FPI and the Navicular Drop. These findings reinforce the evidence of the positive effect of RF in the treatment of FFF in adults.

Several studies conclude that strengthening the intrinsic muscles is more effective than exercises targeting the extrinsic muscles for the treatment of FFF [64,66]. Additionally, it has been shown that muscle-toning exercises yield better results than the use of FO in treating pain associated with flatfoot [20]. Furthermore, exercises have proven effective in improving the MLA and reducing the Navicular Drop [34]. In a recent meta-analysis, Huang et al. [17] concluded that intrinsic muscle exercises significantly normalize foot alignment compared to other interventions, although no significant difference was observed in muscle hypertrophy. Similarly, another recent review on the effectiveness of exercises in treating FFF in adults concluded that strengthening the plantar intrinsic muscles primarily improves gait kinetics and kinematics, corrects foot alignment, and reduces pronation, pain, and disability [66]. All these findings align with those of the present review.

As for the secondary objective of determining which exercises are most effective for pediatric FFF and what the minimum duration of execution is to achieve improvements in signs and symptoms, it is very difficult to reach a consensus due to the wide variety of exercises, protocols, durations, and frequencies used in the 12 articles included in this systematic review [39,40,41,42,43,44,45,46,47,48,49]. We found that the intrinsic muscles are almost always involved in all strengthening or FT exercises. This may be because the intrinsic muscles are one of the main structures that maintain the MLA. The anatomical characteristics of the intrinsic muscles, along with modifications in their mechanical properties, may play a significant role in the onset of FF and the reduction in MLA height [67].

It has been observed that strengthening the TP tendon, as an extrinsic muscle, as well as stretching the triceps surae or Achilles tendon, are recurring components in the different exercise programs described in the articles included in this review [39,40,41,42,43,44,45,46,47,48,49]. In several cases, TP dysfunction originates from a deficit in the function of the navicular–calcaneal-plantar ligament, also known as the spring ligament, which plays a crucial role in MLA stability. Insufficiency of this ligament can lead to increased stress on the posterior tibial tendon, which, already compromised, undergoes progressive fatigue [68]. Additionally, several authors agree that improving stability is a key goal in the treatment of pediatric FF, as muscle strengthening also contributes to improving balance [39]. Although these findings are supported by various studies, the literature has yet to identify a specific exercise or protocol that is considered ideal for managing pediatric FFF [52,55,64].

When comparing exercises or FT with other conservative treatments for pediatric FFF, our review only allows for comparisons with the use of FO combined with exercises [30], taping combined with exercises [41], weight reduction combined with exercise [48], barefoot walking accompanied by exercises [43], and the combination of NMES with exercise [43], versus the exclusive application of exercise. In all the studies analyzed, improvements were significantly greater in the groups that received combined treatment with exercises compared to those who only performed exercises, except in the case of taping combined with exercise, where no significant differences were observed [41]. Based on these findings, it can be concluded that a combined approach of exercise and conservative treatment yields better results in managing pediatric FFF. Regarding the use of taping in treating PFF, although the study included in our review [41] did not report improvements, like other published articles [69,70], other studies in the literature suggest that taping is an effective conservative treatment for PFF [71,72].

Previous research has highlighted considerable ambiguity in the terminology used to describe flexible flatfoot (FFF), with terms such as “valgus flatfoot”, “flatfoot”, and others [9,73]. This issue persists in the current literature, as while most authors refer to it as “flexible flatfoot”, some prefer to classify it as “symptomatic” or “asymptomatic”, others use expressions like “mobile flat feet” or “flexible flatfoot”, and some even categorize it according to different scales. This reflects the lack of consensus in the nomenclature of this condition. This lack of uniformity is also evident when diagnosing FF. Although radiographs are considered the “gold standard” for diagnosing flatfoot [74], their use has been questioned due to ethical concerns related to radiation exposure. Since an accurate diagnosis can be made through clinical tests, and considering the ethical issues associated with radiation, radiographs are not routinely used to diagnose FF. Of all the available clinical tests, the FPI-6 is the only test validated for children over six years old [75], but it is not the most widely used. Instead, the most used test to diagnose pediatric FFF is the NDT, although it presents a considerable issue, as each author establishes different values to classify it as positive or negative. This variability in interpretation contributes to the widespread confusion regarding pediatric FFF, its diagnosis, and its treatment.

Finally, it is important to note that none of the articles included in this review address the use of proprioceptive neuromuscular facilitation (PNF) or hip muscle strengthening, despite both therapies having been shown to be highly useful in treating pediatric FFF [75,76,77,78]. Both hip muscles strengthening and PNF training have shown significant improvements in the stability of the lumbopelvic–hip complex. These interventions may be related to the correction of abnormalities in the lower limb movement pattern, which are common in patients with flatfoot. Both hip muscles strengthening and PNF contributed to improving the stability of the lumbopelvic–hip complex, which could be linked to the existence of movement pattern abnormalities in lower limbs in individuals with FF [78]. The collapse of the MLA could lead to a series of issues along the kinetic chain, such as heel eversion, internal rotation of the tibia, knee valgus, internal rotation of the femur, internal rotation of the hip, and adduction, which may result in anterior pelvic tilt, instability in the lumbopelvic–hip complex, muscle imbalances, joint dysfunctions, and an increased risk of injury [76,78,79]. For this reason, pediatric flatfeet should not be ignored or underestimated. The earlier an effective treatment is administered, the less damage will occur to other parts of the body. When possible, a more conservative corrective procedure should be performed before resorting to irreversible options that may destroy the joints.

This systematic review presents several strengths that should be highlighted. First, we believe it is the first systematic review focused on the use of RF or exercises in the treatment of pediatric FFF. Additionally, no specific filters were applied in the selection of studies; instead, inclusion criteria were strictly followed once all available articles were retrieved, which helped avoid biases in the results. Furthermore, a wide range of databases were consulted, ensuring the thoroughness of the search. The risk of bias is low, and a comprehensive compilation of exercises that can be implemented in the treatment of pediatric FFF is provided.

The main limitation of this study is that seven of the included articles did not have a placebo group, which makes it difficult to make a more precise comparison between the efficacy of exercises and the natural evolution of the foot. Additionally, the average age of the participants is 9 years, which is considered a relatively late stage to observe optimal results with the use of exercises or RF. Another significant limitation was the variation in outcome measures used, along with the heterogeneity observed in the interventions, since nearly every author used a different strengthening methodology. The lack of uniformity in diagnostic methods and inclusion/exclusion criteria limits the comparability between studies, highlighting the need for standardization in pediatric FFF research.

Future research should focus on implementing standardized diagnostic protocols that incorporate validated tests. Additionally, it would be advisable to segment studies into two groups: those with children under 6 years old and those with participants aged 6 or older. It would also be advisable for the children to be younger (between 6 and 8 years old). It would also be valuable to conduct longitudinal follow-up to determine whether the improvements achieved are maintained in the long term. A crucial aspect is that all authors should use the same diagnostic protocol for FFF and employ homogeneous outcome variables, which would allow for stronger and more conclusive evidence. As reported by Vito Pavone et al. [80] in their recent study, it is of great importance to take into account in subsequent studies (age, laxity, diffuse pain, ankle joint pain, Meary’s angle, talonavicular coverage, lateral talocalcaneal angle, and improved ability to walk longer without symptoms). Future research should explore the long-term efficacy of functional rehabilitation exercises, particularly in preventing progression from flexible to rigid flatfoot. Additionally, studies should examine the cost-effectiveness of such interventions compared to orthotics or surgical options.

## 5. Conclusions

RF or exercises, including strengthening, stretching, corrective exercises, plyometrics, and barefoot walking, have been shown to be effective in improving the signs and symptoms of pediatric FFF.

Furthermore, when these exercises are combined with other conservative therapies for treating FFF, such as aerobic exercise for weight reduction, electrotherapy, or the use of orthotic devices (FO), the benefits of RF are significantly enhanced. However, based on the available literature, it is not possible to conclusively determine which exercises are most effective for FFF or the minimum duration required to achieve results, due to the wide variability in the types of exercises and their application modalities (frequency, intensity, etc.).

Nevertheless, based on the studies included in this review, it can be suggested that a minimum RF treatment of one month, with a frequency of at least twice per week and a minimum duration of 30 min per session, could be sufficient to observe clinical results. Additionally, exercises targeting the intrinsic muscles of the foot appear to produce the best results in the treatment of pediatric FFF.

Functional rehabilitation exercises offer a practical, cost-effective, and non-invasive approach for managing PFF in children. Clinicians should consider incorporating these exercises into treatment plans, particularly for mild-to-moderate cases, as part of a broader strategy that includes monitoring and patient education.

## Figures and Tables

**Table 1:** Study characteristics and publication characteristics.

Author, Country (Year of Publication); Evidence Level by SIGN; Age (Years)	Sample Size	Year of Age (Mean/SD); Gender (M/F)	Weight in kg (SD); Height in cm (SD)	BMI kg/m^2^ (SD)	Diagnosis, Symptoms; Previous Treatment
Ketabchi et al., Iran (2024) [40]; II + A; 10–12	CG: 10	10.8 (0.78); (10/0)	40.7 (9.49); 138.8 (7.22)	N/A	FFF, asymptomatic; No
	IG: 12	11 (0.85); (12/0)	42.25 (9.05); 143.1 (6.14)		
Markowicz et al., Poland (2023) [47]; II + A; 7–12	CG: 15	9.3 (1.9); (8/7)	N/A	17.8 (2.4)	Bilateral, symptomatic FFF; No
	IG 1: 15	10.1 (1.8); (9/6)		24.9 (4.0)	
	IG 2: 15	9.4 (1.9); (10/5)		16.9 (1.6)	
Park et al., Republic of Korea (2023) [44]; II + A; 7–8	CG: 9	7.56 (01.77); (6/3)	24.50 (13.96); 122.67 (14.43)	N/A	Pes planus; N/A
	IG: 9	8.56 (02.21); (5/4)	23.28 (07.29); 112.4 (13.49)		
Gheitasi et al., Iran (2022) [46]; II + A; 12–14	CG: 12	13.3 (0.33); N/A	49.3 (2.8); 151(2.67)	21.25 (0.86)	Flat Feet; No
	IG 1: 12	13.3 (0.42); N/A	51.4 (2.7); 159.7(1.39)	20.24 (1.12)	
	IG 2: 12	12.8 (0.61); N/A	52.7 (3.72); 157(3.83)	21.05 (0.93)	
Karthika et al., India (2022) [48]; II + A; 7–14	CG: 12	11.64 (1.38); (6/6)	N/A	28 (1)	FFF; N/A
	IG: 12	11.52 (0.89); (7/5)		27.6 (1.9)	
Rusu et al., Romania (2022) [45]; II + A; 7–11	CG: 15	9.37 (1.42); (17/13)	41.8 (12.72); 148.7 (10.96)	18.84 (5.32)	Bilateral FFF level II, asymptomatic; N/A
	IG:15				
Abd-Elmonem et al., Egypt (2021) [42]; II + A; 7–12	CG: 36	9.45 (0.76); (17/19)	39.27 (6.45); 147.91 (7.69)	17.82 (1.59)	FFF, asymptomatic; N/A
	IG: 36	9.55(1.02); (16/20)	37.9 (8.22); 148 (7.85)	17.12 (2.25)	
Priyanka et al., India (2020) [41]; I-B; 13–14	CG: 22	N/A; N/A	N/A; N/A	N/A	Flat foot; N/A
	IG: 22				
Sativani et al., Indonesia (2020) [49]; I-B; 6–10	CG: 15	8.07 (1.26); (18/12)	N/A; N/A	23 (1.9)	FFF; N/A
	IG: 15				
Sharath Hullumani et al., India (2020) [43]; II + B; 6–14	CG: 19	9.79 (2.07); (11/8)	N/A; N/A	N/A	Flat foot; N/A
	IG: 19	9.42 (2.31); (13/6)			
Khamooshi et al., Iran (2016) [39]; II-B; 9–13	CG: 20	11.5 (0.9); (0/20)	37.9 (2.5); 142.4 (5)	N/A	FFF; No
	IG 1: 20	11.2 (0.8); (0/20)	38.1 (3.4); 142.9 (5)		
	IG 2: 20	11.18 (0.8); (0/20)	36.7 (3.4); 143.4 (5)		

Abbreviations: SIGN: Scottish Intercollegiate Guidelines Network; IG: intervention group; CG: control group; N/A: Not Available; SD: standard deviation; M: male; F: female; BMI: body mass index; FFF: flexible flat feet.

**Table 2:** Sample selection and diagnoses.

Sample Selection			Diagnoses
Authors	Inclusion Criteria	Exclusion Criteria	PFF Definition and Assessment
Ketabchi et al. (2024) [40]	Asymptomatic FFF	- Leg length discrepancies - Previous foot or leg surgery or injury - Diagnosis of rigid flat foot - Tarsal coalitions - Current use of insoles - Congenital defects of lower limbs	Orthopedist-diagnosed: asymptomatic FFF NDT ≥ 10 mm
Markowicz et al. (2023) [47]	Children with bilateral, symptomatic FFF	- Tarsal coalitions - Congenital defects of lower limbs - Neurological diseases - Previous foot surgery - Girls who have not started their menstrual cycle	Orthopedist-diagnosed: asymptomatic FFF Physiotherapy examination FPI-6 Postural Stability Test (Biodex balance system SD 115VAC)
Park et al. (2023) [44]	- Children diagnosed with developmental delay - Grade ≤ 1 on podoscope examination and without changes in the examination results for 1 year - Children who can follow instructions and who have been walking independently for more than 1 year	- Children who cannot follow instructions - Children who have spasticity over MAS 1 in lower extremities	Grade 1, 2 or 3 on podoscope Arch height index (was compared using ImageJ) Gait parameters with footprints (Gait velocity (cm/s); Step length (cm); Stride length (cm); Step Angle (°) and the width of the foot (cm))
Gheitasi et al. (2022) [46]	- Subjects with flat feet - Healthy weight - No previous treatment	- Neurological, orthopedic, or cardiovascular diseases - Abnormal BMI - Surgical trauma, foot fracture in the last 6 months - Not willing to participate	Fatigue earlier than usual Deficit in foot function Risk of pain Dysfunction in ankle, knee, and hip joints NDT > 10 mm
Karthika et al. (2022) [48]	- FFF level II, asymptomatic - Subjects with FFF - BMI between 25 and 30 - Grade 2 and 3 in foot structure assessment	- Foot pain - History of lower limb injury (6 months) - Congenital foot or leg abnormalities - Unequal lower limb - Rigid pes planus	NDT FPI-6
Rusu et al. (2022) [45]	- FFF after static and dynamic analysis	- Foot or ankle surgery - Lower limb pain - Overweight - Neuromuscular or neurological disorders	Clinical examination in standing and walking Arch height index and subtalar flexibility, which was assessed by a force platform
Abd-Elmonem et al. (2021) [42]	- Diagnosed asymptomatic FFF - Grade III flatfoot classification	- Congenital deformities of the lower limb - Scar/osseous anomalies	Diagnosed by an orthopedist NDT > 9 mm
Priyanka et al. (2020) [41]	Children with flat feet	- Previous trauma or fracture of lower limb - History of previous surgeries of the lower limb during the last 3 months - Hypersensitive skin, and any allergy to tape	“Too many toes” sign RCSP > 10° Navicular height (Less than 1 cm)
Sativani et al. (2020) [49]	- FFF	- Ankle fracture - Painkillers	NDT > 10 mm Reduced medial longitudinal arch height
Sharath Hullumani et al. (2020) [43]	Flat foot	- Musculoskeletal injuries (6 months) - Mental disorders - Limb length discrepancy	Arch Index was used to check the distance of the ankle axis
Khamooshi et al. (2016) [39]	- FFF - 9–13 years of age - Female - Good health status	- Previous lower extremities surgery or lesion - Severe orthopedic problems	Staheli index (Pedoscope and foot arch rate) Positive navicular collapse test

Abbreviations: FFF: Flexible flat feet; BMI: Body mass index; MAS: Modified Ashworth Scale; FPI: Foot posture index; NDT: Navicular drop test; RCSP: Resting calcaneal stance position.

## Supplementary Materials

The following supporting information can be downloaded at: https://www.mdpi.com/article/10.3390/children12010008/s1, Table S1: PRISMA checklist.
