# Supplementary material for: Efficacy of Functional Re-Education as a Treatment for Infantile Flexible Flatfoot: Systematic Review
Source: Children (Basel). 2024 Dec 24;12(1):8. doi: 10.3390/children12010008 (PMC11763734; doi:10.3390/children12010008)
Supplement: Supplementary file 1 [file children-12-00008-s001.zip › children-3348069-supplementary.pdf]

PRISMA 2020 Checklist

Section and Topic	Item #	Checklist item	Location where item is reported
**TITLE**			
Title	1	Identify the report as a systematic review.	Lines 2-3: *Efficacy of functional re-education as a treatment for infantile flexible flatfoot. Systematic Review.*
**ABSTRACT**			
Abstract	2	See the PRISMA 2020 for Abstracts checklist.	Lines 18-34: Structured abstract including background, objectives, methods, results, and conclusion.
**INTRODUCTION**			
Rationale	3	Describe the rationale for the review in the context of existing knowledge.	Lines 38-112: Context and background about the importance of treating pediatric flatfoot.
Objectives	4	Provide an explicit statement of the objective(s) or question(s) the review addresses.	Lines 102-108: *This systematic review aims to evaluate the effectiveness of functional re-education.*
**METHODS**			
Eligibility criteria	5	Specify the inclusion and exclusion criteria for the review and how studies were grouped for the syntheses.	Lines 122-148: Details on inclusion and exclusion criteria, and data grouping.
Information sources	6	Specify all databases, registers, websites, organisations, reference lists and other sources searched or consulted to identify studies. Specify the date when each source was last searched or consulted.	Lines 154-154: Databases consulted (PubMed, Cochrane, SCOPUS, etc.). Last search conducted in September 2024.
Search strategy	7	Present the full search strategies for all databases, registers and websites, including any filters and limits used.	Lines 160-172: Search strategy with MeSH terms and Boolean operators.
Selection process	8	Specify the methods used to decide whether a study met the inclusion criteria of the review, including how many reviewers screened each record and each report retrieved, whether they worked independently, and if applicable, details of automation tools used in the process.	Lines 174-180: Selection process by independent reviewers and resolution of discrepancies.
Data collection process	9	Specify the methods used to collect data from reports, including how many reviewers collected data from each report, whether they worked independently, any processes for obtaining or confirming data from study investigators, and if applicable, details of automation tools used in the process.	Lines 174-180: Data extraction and management performed by two independent reviewers.
Data items	10	List and define all outcomes for which data were sought. Specify whether all results that were compatible with each outcome domain in each study were sought (e.g. for all measures, time points, analyses), and if not, the methods used to decide which results to collect.<br>List and define all other variables for which data were sought (e.g. participant and intervention characteristics, funding sources). Describe any assumptions made about any missing or unclear information.	Lines 182-189: *Outcome measures included MLA formation, symptom reduction, and test scores.* Extracted information on interventions, participants, and results.
Study risk of bias assessment	11	Specify the methods used to assess risk of bias in the included studies, including details of the tool(s) used, how many reviewers assessed each study and whether they worked independently, and if applicable, details of automation tools used in the process.	Lines 191-198: Cochrane and SIGN tools used to assess bias.
Effect measures	12	Specify for each outcome the effect measure(s) (e.g. risk ratio, mean difference) used in the synthesis or presentation of results.	Lines 199-203: No meta-analysis conducted; results analyzed narratively.

PRISMA 2020 Checklist

Section and Topic	Item #	Checklist item	Location where item is reported
Synthesis methods	13	Describe the processes used to decide which studies were eligible for each synthesis (e.g. tabulating the study intervention characteristics and comparing against the planned groups for each synthesis (item #5)).<br>Describe any methods required to prepare the data for presentation or synthesis, such as handling of missing summary statistics, or data conversions.<br>Describe any methods used to tabulate or visually display results of individual studies and syntheses.<br>Describe any methods used to synthesize results and provide a rationale for the choice(s). If meta-analysis was performed, describe the model(s), method(s) to identify the presence and extent of statistical heterogeneity, and software package(s) used.<br>Describe any methods used to explore possible causes of heterogeneity among study results (e.g. subgroup analysis, meta-regression).<br>Describe any sensitivity analyses conducted to assess robustness of the synthesized results.	Table of characteristics and narrative analysis; meta-analysis not feasible.<br>Results tabulated or described narratively.<br>Figures and tables (e.g., PRISMA flow diagram).<br>Narratives based on Cochrane Collaboration recommendations.
Reporting bias assessment	14	Describe any methods used to assess risk of bias due to missing results in a synthesis (arising from reporting biases).	Not applicable.
Certainty assessment	15	Describe any methods used to assess certainty (or confidence) in the body of evidence for an outcome.	Line 191: Bias assessment as part of methodological quality.
**RESULTS**			
Study selection	16	Describe the results of the search and selection process, from the number of records identified in the search to the number of studies included in the review, ideally using a flow diagram.<br>Cite studies that might appear to meet the inclusion criteria, but which were excluded, and explain why they were excluded.	Lines 205-218: Process described with PRISMA diagram. Excluded studies mentioned (total number and general reasons).
Study characteristics	17	Cite each included study and present its characteristics.	Lines 249-250: Details on included studies (Table 1).
Risk of bias in studies	18	Present assessments of risk of bias for each included study.	Lines 293-295: Figure 2 (risk of bias).
Results of individual studies	19	For all outcomes, present, for each study: (a) summary statistics for each group (where appropriate) and (b) an effect estimate and its precision (e.g. confidence/credible interval), ideally using structured tables or plots.	Lines 217-229: Individual results summarized for each group of studies.
Results of syntheses	20	For each synthesis, briefly summarise the characteristics and risk of bias among contributing studies.<br>Present results of all statistical syntheses conducted. If meta-analysis was done, present for each the summary estimate and its precision (e.g. confidence/credible interval) and measures of statistical heterogeneity. If comparing groups, describe the direction of the effect.<br>Present results of all investigations of possible causes of heterogeneity among study results.<br>Present results of all sensitivity analyses conducted to assess the robustness of the synthesized results.	Line 220-295: Summary analysis of bias and general study characteristics.<br>Not applicable (no meta-analysis conducted).
Reporting	21	Present assessments of risk of bias due to missing results (arising from reporting biases) for	Not applicable.

PRISMA 2020 Checklist

Section and Topic	Item #	Checklist item	Location where item is reported
biases		each synthesis assessed.	
Certainty of evidence	22	Present assessments of certainty (or confidence) in the body of evidence for each outcome assessed.	Mentioned as part of bias context (lines 288-292).
**DISCUSSION**			
Discussion	23	Provide a general interpretation of the results in the context of other evidence.<br>Discuss any limitations of the evidence included in the review.<br>Discuss any limitations of the review processes used.<br>Discuss implications of the results for practice, policy, and future research.	Lines 400-524: General discussion of findings.<br>Lines 525-533: Limitations of included studies and related to the review methodology.<br>Lines 535-541: Recommendations for future research.
**OTHER INFORMATION**			
Registration and protocol	24	Provide registration information for the review, including register name and registration number, or state that the review was not registered.<br>Indicate where the review protocol can be accessed, or state that a protocol was not prepared.<br>Describe and explain any amendments to information provided at registration or in the protocol.	Line 115: Registered in PROSPERO (CRD42023391030).
Support	25	Describe sources of financial or non-financial support for the review, and the role of the funders or sponsors in the review.	Line 566: *This research received no external funding.*
Competing interests	26	Declare any competing interests of review authors.	Line 571: *The authors declare no conflicts of interest.*
Availability of data, code and other materials	27	Report which of the following are publicly available and where they can be found: template data collection forms; data extracted from included studies; data used for all analyses; analytic code; any other materials used in the review.	Line 569: *No new data were created or analyzed in this study.*

From: Page MJ, McKenzie JE, Bossuyt PM, Boutron I, Hoffmann TC, Mulrow CD, et al. The PRISMA 2020 statement: an updated guideline for reporting systematic reviews. BMJ 2021;372:n71. doi: 10.1136/bmj.n71. This work is licensed under CC BY 4.0. To view a copy of this license, visit https://creativecommons.org/licenses/by/4.0/
